# Patient‐reported outcomes of laryngeal mask anesthesia in thoracoscopic pulmonary wedge resection: A randomized controlled study

**DOI:** 10.1111/1759-7714.14675

**Published:** 2022-09-30

**Authors:** Kaikai Xu, Yi Zhang, Yong Cui, Feng Tian

**Affiliations:** ^1^ Department of Thoracic Surgery Xuanwu Hospital Capital Medical University Beijing China; ^2^ Department of Thoracic surgery Capital Medical University Affiliated Beijing Friendship Hospital Beijing China

**Keywords:** endotracheal intubation of double lumen tube, laryngeal mask, patient‐reported outcomes, randomized controlled trial, thoracoscopic pulmonary wedge resection

## Abstract

**Background:**

To assess the effectiveness and safety of laryngeal mask anesthesia (LMA) in thoracoscopic pulmonary wedge resection based on patient‐reported outcomes (PROs).

**Methods:**

This randomized controlled trial included 107 patients who underwent thoracoscopic pulmonary wedge resection between June 2017 and December 2021 for pulmonary nodule or pulmonary bullae. In one group, LMA was applied for general anesthesia, and in the other group, endotracheal intubation (ETT) was used.

**Results:**

A total of 107 patients were included in the study. The symptom assessment based on PROs showed that the incidence of pharyngodynia, trachyphonia, and cough were lower in the LMA group, while the postoperative gastrointestinal reaction did not significantly differ between the two groups. The pain score and global satisfaction score were significantly better in the LMA group. The satisfaction degree of anesthesia and the surgical field did not significantly differ between the two groups. The anesthesia recovery time, indwelling days of chest catheter, and postoperative hospital stay of the LMA group were all shorter, while the operation time, intraoperative blood loss and lowest intraoperative oxygen saturation did not significantly differ between the two groups. The highest intraoperative partial pressure of CO_2_ was significantly higher in the LMA group. The artery blood gas analysis after the operation did not significantly differ between the two groups.

**Conclusion:**

Compared with ETT, the application of LMA may demonstrate promising advantages in airway management for thoracoscopic pulmonary wedge resection.

Registration No. of clinical trial (ChiCTR2000034905).

## Graphical abstract

Which is better anesthesia for thoracoscopic pulmonary wedge resection, LMA or ETT?

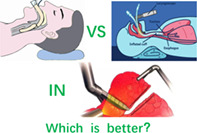

## INTRODUCTION

Enhanced recovery after surgery (ERAS) has become one of the mainstream targets in thoracic surgery departments, and the optimization of the anesthesia method is an important component in the processes of ERAS.^1^ Currently, the mainstream anesthesia method used in thoracic surgery departments is the endotracheal intubation of a double‐lumen tube or implantation of a bronchial plug under general anesthesia and contralateral one‐lung ventilation (OLV). Although these methods can effectively achieve pulmonary isolation, they can also induce various endotracheal intubation‐related complications, such as pulmonary infection, ventilation pressure‐induced pulmonary injury, over‐distention‐induced pulmonary injury, bronchospasm, impairment of cardiac function, arrhythmia, postoperative pharyngalgia, and irritable cough.^2^ To address these issues, nonendotracheal intubation anesthesia^2^ has become more extensively applied in thoracoscopic surgeries. Laryngeal mask anesthesia (LMA), which utilizes the laryngeal mask for ventilation and preserves autonomous respiration, has several advantages, including easy performance, short time, fewer complications, fast recovery, and low costs.^3^ Therefore, LMA has become more extensively applied in surgeries in thoracic surgery departments.

With the application of relatively mature ERAS processes, the incidence of perioperative complications in thoracoscopic pulmonary wedge resection can be kept at a low level, which then makes symptom management and subjective feeling improvement of patients especially important. Currently, the positive effects of clinical application of ERAS mainly include the reduced incidence of perioperative complications and shorter hospital stays. However, some researchers^4^ suggested that the effects of ERAS should be assessed based on doctors' views, placing less emphasis on perioperative symptom management and the quality of life of patients. Therefore, patient‐reported outcomes (PROs) have been suggested as a relevant indicator for the assessment of ERAS. By utilizing interviews, self‐rating scales, and other data collection tools, PROs can directly acquire data reported by patients, thus providing a measurement for assessing the treatment effects in clinical practice.^4^ However, thus far, only a few studies on the clinical application of LMA investigated patients' symptoms and subjective feelings by using PROs. In addition, the high‐grade evidence on the safety and effectiveness of LMA in thoracic surgery departments is limited, and only a few studies compared LMA with endotracheal intubation anesthesia. Therefore, this randomized controlled trial was designed to assess the effects of LMA in thoracoscopic pulmonary wedge resection based on PROs, and to further assess the effectiveness and safety of LMA.

## METHODS

### Study design and subjects

This study was a single‐center, open‐label, prospective, randomized controlled trial. It was reviewed and approved by the Ethics Committee of Beijing Friendship Hospital, Capital Medical University (Ethics approval no. 2016‐P2‐045‐02).

Between June 2017 and December 2021, patients diagnosed with pulmonary nodules or pulmonary bullae and approved for thoracoscopic pulmonary wedge resection were included after discussion in the department. The inclusion criteria were as follows: (1) Aged 18–70 years, (2) anesthesia risk score of grade I–II, (3) Mallampati airway grade I–II, and (4) the patients who signed informed consents. The exclusion criteria were: (1) A history of respiratory diseases, such as tuberculosis, chronic obstructive pulmonary diseases (COPD), and sleep apnea‐hypopnea syndrome (SAHS); (2) with a history of circulatory system diseases (such as severe coronary heart disease and rheumatic heart disease), or diseases of surgical contraindications, such as dysfunctions of other vital organs and coagulation dysfunctions; (3) imaging examinations which showed evident ipsilateral pleural adhesions; (4) with contraindications of LMA or general anesthesia by endotracheal intubation of double‐lumen tube; (5) with mental disturbances and those not able to cooperate with the anesthesia or surgery; and (6) BMI ≥30 kg/m^2^.

For each patient, a randomization code was generated by the IBM SPSS Statistics version 24.0 software (IBM), based on which the patients were randomized by the simple randomization method into the LMA group and double‐lumen tube anesthesia group (ETT group). The grouping information was kept in the form of codes by an independent third‐party responsible for data analysis. Patients in the LMA group underwent laryngeal mask anesthesia, and patients in the ETT group underwent general anesthesia by endotracheal intubation of a double‐lumen tube. Based on “rule of thumb” in statistics and a previously published article,^5^ at least 50 patients in each group were considered adequate for statistical analysis. Patients were recruited until the sample size for each group reached the ideal number.

### Anesthesia method

For LMA: patients were placed in 30° semi‐reclining position. A fiberoptic bronchoscope, equipment for tracheal intubation, and bronchial plug were prepared before anesthesia, so tracheal intubation could be immediately performed if necessary. Continuous anesthesia monitoring was performed for all the patients, including electrocardiograph (ECG), heart rate, pulse blood oxygen saturation (SpO2), respiratory rate (RR), and continuous invasive arterial blood pressure (IABP) by radial artery puncture, and bispectral index (BIS). After intravenous pumping of a loading dose (1 μg/kg), dexmedetomidine hydrochloride within 10 min was performed, the pumping was stopped, and intravenous anesthesia induction was performed. Intravenous injection of propofol (2.5 mg/kg) and sufentanil (0.1 μg/kg) was given. After the lower mandible relaxed, a BlockBuster laryngeal mask was implanted (size 4 laryngeal mask for males and size 3 laryngeal mask for females). In patients with difficult implantation of the laryngeal mask, an intravenous injection of 0.5 mg/kg propofol was given again, followed by another implantation of the laryngeal mask. Next, inhalation of sevoflurane for anesthesia maintenance and pure oxygen inhalation with the oxygen flow of 2 l/min were given. The inhalation dose of sevoflurane was adjusted to maintain the BIS value between 40 and 60. The oxygen saturation was maintained at ≥90% during the operation procedures. Next, an incision was made at the lateral thoracic wall to access the thoracic cavity, which also induced iatrogenic pneumothorax that led to the gradual collapse of the ipsilateral lung, and equipment was used for auxiliary squeezing of pulmonary lobes. When the surgery was completed, the laryngeal mask was removed after the patients had awoken, and had been transferred to the anesthesia recovery room for further monitoring.

For endotracheal intubation anesthesia: patients were placed in the supine position, and anesthesia monitoring was performed as in the LMA group. After intravenous pumping of a loading dose (1 μg/kg), dexmedetomidine hydrochloride within 10 min was performed, the pumping was stopped, and intravenous anesthesia induction was performed. Then, 2 mg/kg propofol, 0.3 mg/kg sufentanil, and 0.6 mg/kg rocuronium bromide were used for anesthesia induction. The left endotracheal double‐lumen tube was intubated after complete muscle relaxation. During OLV, the mechanical ventilation parameter was set at 6–8 mg/kg, the inspiratory/expiratory ratio was 1:1–1:2, the concentration of end‐tidal CO_2_ (CETO_2_) was maintained at 35–45 mmHg, and peak airway pressure was maintained at <20 cmH_2_O. Inhalation of sevoflurane was performed for anesthesia maintenance, and pure oxygen inhalation with the oxygen flow of 2 l/min was performed. Continuous intravenous pumping of 0.1–0.3 μg/kg/min remifentanil was performed to maintain the BIS value between 40 and 60. After surgery was completed and the patient awoke, the endotracheal double‐lumen tube was removed, and the patient was transferred to the anesthesia recovery room for further monitoring.

A paravertebral block (TPB) was performed for patients in both groups after anesthesia was completed but before the surgical procedures. In brief, the patients were placed in the lateral position, after which TPB of the fifth thoracic vertebra was performed under the guidance of ultrasound. After the puncture succeeded, 0.5% ropivacaine and 10 mg dexamethasone with a total volume of 20 ml were injected.

### Surgical procedures and postoperative management

For patients in both groups, single hole thoracoscopic surgery was performed. The incision was generally made at the anterior axillary line at the fourth or fifth intercostal space. The thoracic wall was incised layer by layer to access the thoracic cavity, and an incision protector was placed. The probe of the thoracoscope and equipment were inserted for exploration. For patients with a pleural adhesion, an electrocoagulation hook or ultrasonic scalpel was used to separate the adhesion under thoracoscopy, thus providing a good visual field and operating space. Next, the site of pulmonary nodules or pulmonary bullae was clarified, and an endoscopic cut and suture device were used to resect the tissues with the lesion. After the procedures were completed, the thoracic cavity was rinsed, then the anesthesiologist inflated the lungs to check for lung leakage. The lung inflation methods of two groups were the same. A thoracic drainage tube was placed. When the postoperative closed drainage of the thoracic cavity showed no air leakage and 24 h drainage volume <100 ml, the drainage tube was removed.

### Endpoints

For all the patients, the results of preoperative anesthesia (successful or not) were recorded. Intraoperative conversion to endotracheal intubation, conversion to thoracotomy, and death were also recorded.

#### Primary endpoints

According to the symptom assessment criteria described in previous studies and guidelines, a comprehensive patient symptom and satisfaction degree assessment scale was developed. The assessments of all patients were performed on the day of hospital discharge. The symptom and satisfaction degree assessment scale was completed by patients under the guidance of two trained doctors.

1. Gastrointestinal reaction: the postoperative gastrointestinal reactions of the patients were assessed according to the criteria described in the fourth edition of *Guidelines for the Management of Postoperative Nausea and Vomiting*.^6^ In brief, grade 0 indicated no symptoms of gastrointestinal reaction; grade I indicated dizziness but no nausea or retching; grade II indicated mild nausea but no vomiting that was accompanied by abdominal discomfort; grade III indicated evident nausea that was accompanied by retching but with no content vomited; grade IV indicated severe vomiting symptoms, where the vomiting of gastric juice and contents could not be controlled by drugs.
2. Hoarseness: with/without the symptom of hoarseness.
3. Pharyngalgia and pharyngeal discomfort: the Prince‐Henry scoring system^7^ was used for assessment, and the criteria were as follows: grade 0, no pharyngalgia or pharyngeal discomfort; grade I, no pain in waking state, but with pain during cough; grade II, with evident pain during deep breathing; grade III, with endurable pain in waking state; and grade IV, with drastic pain in resting‐state that could not be tolerated.
4. Cough: the Mandarin Chinese version Leicester Cough Questionnaire (LCQ‐MC)^8^ was used to assess postoperative coughing in patients. LCQ‐MC included 19 questions in three dimensions, that is, physiology, psychology, and social dimensions. Each question included seven choices (positive scoring, 1–7 points, with the higher scores indicating milder coughing symptoms). The average score of the questions in the dimension was calculated, which was considered as the score of the dimension (1–7 points). The total score was calculated by adding up the scores of the three dimensions (3–21 points).
5. Global pain score: the visual analogue scale was used to assess postoperative pain. The symptoms were rated on an 11‐point scale, with 0 indicating “not present”, and 10 indicating “as bad as you can imagine.”
6. Global satisfaction score: the visual analogue scale was used for the assessment of the feelings of overall surgical procedures and satisfaction degree. The degree of satisfaction was rated on an 11‐point scale, with 0 indicating “highly satisfactory”, and 10 indicating “as bad as you can imagine.”

#### Secondary endpoints

Intraoperative satisfaction degree with anesthesia and satisfaction degree with surgical field: immediately after the surgical procedures were completed, the satisfaction degrees with anesthesia and surgical field were respectively assessed by the anesthetists and operators. The criteria^9^ were as follows: (1) rating satisfaction degree of anesthesia: level 1, perfect anesthesia, where the patients were painless and quiet, which provided good conditions for surgery; the hemodynamics were generally stable; level 2, suboptimal anesthesia, patients had mild pain manifestations, muscle relaxation was suboptimal and required sedatives; there were variations in hemodynamics (not induced by disease conditions); level 3, imperfect anesthesia, the patient experienced substantial pain, or the muscle relaxation effect was poor and groaning, or agitation were common; after auxiliary drug therapy, the situation improved but was still suboptimal, barely allowing the doctors to complete the surgery; level 4, other anesthesia was required to complete the surgery. (2) Rating of satisfaction degree with surgical field: level 1, the exposure to the surgical field was satisfactory; level 2, the surgical field was generally clear, the lung collapse was fair, but there was no need to stop the surgery; level 3, the surgical field exposure was relatively poor, lung collapse was unsatisfactory, and surgery was interrupted most of the time; and level 4, the surgical field exposure was very poor, and the surgery could not be performed; thus, it was necessary to convert to endotracheal intubation.

All the following clinical data were collected from the anesthesia and surgical notes and the medical and nursing records. The collected data included: time of operation, intraoperative blood loss, lowest intraoperative oxygen saturation and the peak of end‐tidal CO_2_ pressure, duration of laryngeal mask and endotracheal tube placement，anesthesia recovery time, blood gas analysis 1 h after the operation, and postoperative indwelling days of chest catheter, and hospital stay.

### Statistical analysis

Under the guidance of two trained doctors, patients completed the assessment of symptoms and feelings on the day of hospital discharge. The objective data were collected from the anesthesia, medical, and nursing records. Quantitative data were described by means and standard deviations, and a *t*‐test was used for the comparisons between groups. Categorical data were analyzed by the Chi‐square test. All data were analyzed by using the SPSS 24.0 software (IBM). All statistical analyses were two‐sided, and *p* < 0.05 was considered statistically significant.

## RESULTS

### Clinical data

A total of 107 adult patients, 53 in the LMA group and 54 in the ETT group, who underwent thoracoscopic pulmonary wedge resection in the indicated time, were included in this study. The diseases in the patients included primary lung cancer, metastatic lung cancer, benign pulmonary nodule, and pulmonary bulla. The same surgical team performed all the surgeries. After randomized grouping, there was no significant difference between the LMA and the ETT groups in terms of the physiological parameters and population features of the enrolled patients (Table 1). The implantation of a laryngeal mask/double lumen tube in both groups was successful. The anesthesia and surgeries were successfully completed in all the patients. No intraoperative conversion to endotracheal intubation, conversion to thoracotomy, or death occurred. No other major complications occurred.

**TABLE 1:** Clinical parameters of the enrolled patients

Variables	LMA group *n* = 53	ETT group *n* = 54	*p*‐value
Gender			0.557
Male	33	30	
Female	20	24	
Age (years)	51.02 ± 19.27	50.48 ± 16.30	0.876
Smoker			0.441
Yes	27	23	
No	26	31	
Anesthesia risk score of grade^a^			0.846
I	23	22	
II	30	32	
Mallampati airway grade^b^			0.488
I	43	40	
II	10	14	
BMI			0.281
Normal	32	27	
Abnormal	21	27	
Admitting diagnosis			0.511
Pulmonary nodules	38	42	
Pulmonary bulla	15	12	

*Note*: Continuous data are shown as the mean ± standard deviation.

Abbreviations: BMI, body mass index; ETT, endotracheal intubation; LMA, laryngeal mask airway.

^a^ Risk classification of anesthesia of American Society of Anesthesiologists.

^b^ Modified Mallampati score of airway classification.

### PROs

The assessment of postoperative symptoms of patients showed that the incidence of gastrointestinal reactions was lower in the LMA group than in the ETT group, but the difference was not statistically significant (*p* = 0.130). The data of trachyphonia (*p* = 0.042), pharyngodynia (*p* = 0.001), and cough (*p* = 0.027) were significantly better in the LMA group than in the ETT group, which was manifested by the lower incidence and severity. The perioperative global pain score (*p* = 0.045) and satisfaction degree score of patients were both better in the LMA group than in the ETT group (Table 2).

**TABLE 2:** PROs score

Variables	LMA group *n* = 53	ETT group *n* = 54	*p* value
Gastrointestinal reaction			0.130
Grade 0	44	36	
Grade I	7	16	
Grade II	2	1	
Grade III	0	1	
Grade IV	0	0	
Hoarseness			0.042
Yes	3	10	
No	50	44	
Pharyngalgia and pharyngeal discomfort			0.001
Grade 0	47	29	
Grade I	6	20	
Grade II	0	4	
Grade III	0	1	
Grade IV	0	0	
Cough			
Social dimension	6.04 ± 0.73	6.11 ± 0.77	0.615
Physiology dimension	5.85 ± 0.68	5.39 ± 0.76	0.001
Psychology dimension	5.75 ± 0.68	5.35 ± 0.73	0.004
Total score	17.64 ± 1.90	16.85 ± 1.73	0.027
Global pain score	2.42 ± 1.10	2.85 ± 1.12	0.045
Global satisfaction score	1.11 ± 0.32	1.35 ± 0.55	0.008

*Note*: Continuous data are shown as the mean ± standard deviation.

Abbreviations: ETT, endotracheal intubation; LMA, laryngeal mask airway.

### Comparison of satisfaction degree to anesthesia and surgical field

The satisfaction degrees with anesthesia and surgical field were assessed respectively by anesthetists and operators immediately after the surgery was completed. No level 3 or 4 satisfaction degree with anesthesia or surgical field was reported in either group. Both anesthesia methods provided satisfactory anesthesia effects and surgical fields that allowed for a successful surgery. Statistical analysis showed that the satisfaction degrees with anesthesia (*p* = 0.139) and surgical field (*p* = 0.511) did not significantly differ between the two groups (Table 3).

**TABLE 3:** Satisfaction degrees of anesthesia and surgical field

Variables	LMA group *n* = 53	ETT group *n* = 54	*p*‐value
Satisfaction degrees of anesthesia			0.139
Level 1	30	38	
Level 2	23	16	
Level 3	0	0	
Level 4	0	0	
Satisfaction degrees of surgical field			0.511
Level 1	38	42	
Level 2	14	12	
Level 3	1	0	
Level 4	0	0	

Abbreviations: LMA, laryngeal mask airway; ETT, endotracheal intubation.

### Intra‐ and postoperative objective endpoints

The operation time (*p* = 0.302) and intraoperative blood loss (*p* = 0.397) did not significantly differ between the two groups. The lowest intraoperative SpO_2_ (*p* = 0.253) was also not significantly different between the two groups. The highest end‐tidal partial pressure of CO_2_ (*p* < 0.001) was significantly higher in the LMA group than ETT group. The time of laryngeal mask placement was significantly shorter than endotracheal tube (*p* < 0.001). The anesthesia recovery time (*p* < 0.001) was significantly shorter in the LMA group than ETT group. The partial oxygen pressure (*p* = 0.641) and partial CO_2_ pressure (*p* = 0.656) at 1 h after the operation did not significantly differ between the two groups. The differences in white blood cells (*p* = 0.244) and neutrophils (*p* = 0.876) before and after operation in the two groups were not statistically significant. The postoperative indwelling days of chest catheter (*p* = 0.040) and postoperative hospital stay (*p* = 0.032) were all shorter in the LMA group than in the ETT group (Table 4).

**TABLE 4:** Intra‐ and postoperative clinical data

Variables	LMA group *n* = 53	ETT group *n* = 54	*p*‐value
Time of operation (min)^a^	60.75 ± 20.99	57.12 ± 14.65	0.302
Intraoperative blood loss (ml)	10.36 ± 14.42	12.69 ± 13.85	0.397
Lowest oxygen saturation (%)^b^	96.91 ± 2.03	94.74 ± 13.56	0.253
PetCO_2_ (mmHg)^c^	51.89 ± 9.54	43.19 ± 5.92	<0.001
Time of laryngeal mask or endotracheal tube placement(s)	42.6 ± 9.22	162.5 ± 40.73	<0.001
Anesthesia recovery time (min)^d^	10.38 ± 2.22	19.04 ± 4.62	<0.001
PaO_2_ (1 h after surgery, mmHg)	82.77 ± 6.43	83.52 ± 9.68	0.641
PaCO_2_ (1 h after surgery, mmHg)	44.64 ± 3.41	44.96 ± 3.99	0.656
△WBC^e^	5.24 ± 2.91	4.66 ± 2.21	0.244
△NEU%^f^	21.76 ± 6.35	21.59 ± 5.17	0.876
Postoperative indwelling days of chest catheter^g^	2.13 ± 0.92	2.48 ± 0.82	0.040
Patients with pulmonary bullae^h^	2.13 ± 1.19	3.00 ± 1.13	0.065
Patients with pulmonary nodules^i^	2.13 ± 0.82	2.33 ± 0.65	0.221
Hospital stay (days)	3.20 ± 0.94	3.57 ± 0.79	0.032

*Note*: Continuous data are shown as the mean ± standard deviation.

Abbreviations: ETT, endotracheal intubation; LMA, laryngeal mask airway.

^a^ From the time of skin incision to the end of suture incision.

^b^ Lowest intraoperative oxygen saturation.

^c^ Peak of intraoperative end‐expiratory pCO_2_.

^d^ The duration time is from when the operation is finished to the time when patients completely recover consciousness, and the time of extubation/ laryngeal mask removed.

^e^ Difference in blood white cell counts (ΔWBC) and percentage of neutrophil granulocytes (ΔNEU%) between the day before surgery and 1 day after surgery.

^f^ Postoperative duration of thoracic drainage.

^g^ Postoperative indwelling days of chest catheter of all patients.

^h^ Postoperative indwelling days of chest catheter of patients with pulmonary bullae.

^i^ Postoperative indwelling days of chest catheter of patients with pulmonary nodules.

## DISCUSSION

Nonendotracheal intubation anesthesia is a “minimally invasive anesthesia” developed according to the requirements of ERAS and extensively applied in thoracoscopic surgeries to meet the target of “integrated minimally invasive anesthesia”.^1^, ^10^, ^11^ LMA method is based on general intravenous anesthesia. It may use a low‐dose muscle relaxant (or not used), while sedatives and analgesics are used as auxiliary means. The laryngeal mask is orally implanted for ventilation. Low tidal volume and high‐frequency ventilation are generally used in the processes, and ipsilateral lung collapse is induced by iatrogenic pneumothorax after accessing the thoracic cavity. When treating key sites such as veins, arteries, and bronchi, additional drugs can be used to reduce the respiratory rate, and TPB or an intercostal nerve block can also be used to help analgesia.^12^ Ambrogi et al.^13^ reported that thoracoscopic surgeries under LMA successfully treat spontaneous pneumothorax. Gonzalez‐Riva et al.^14^ also reported successful thoracoscopic lobectomy under LMA in one case. Many researchers have been using LMA in thoracoscopic surgeries with good effect.

With the advancement in medical techniques and continuous improvements of medical models, patients also have higher requirements given their quality of life. The requirements also continuously remind the doctors that subjective feelings are as important as objective clinical indicators in evaluating clinical efficacies. Therefore, evaluating the clinical efficacies from multidimensions and multilevels, such as clinical manifestations, mental state, social lives, satisfaction degree to self‐health status, and satisfaction degree with clinical efficacy, has attracted increasing attention. PROs are an assessment method that captures the patient's health‐related concepts, which could provide a valid measurement for assessing clinical treatment effects.^15^, ^16^ Thoracoscopic pulmonary wedge resection is a nonanatomical sublobectomy method that has been extensively used in treating pulmonary bullae, benign pulmonary nodule, preinvasive pulmonary adenocarcinoma, and metastatic pulmonary cancer. Currently, thoracoscopic pulmonary wedge resection is performed in most centers. Under the background of mature ERAS processes, the surgical complications could be controlled at extremely low levels. Therefore, improving patient symptoms and subjective feelings in treating such patients is especially important.

In this study, the currently available anesthesia and intraoperative analgesic methods were used in combination. Specifically, LMA that preserves autonomous respiration was used in combination with TPB, after which single‐hole thoracoscopic surgery was performed. Our findings demonstrated that compared with the general anesthesia by endotracheal intubation of a double lumen tube, the postoperative assessment of common symptoms, such as pharyngalgia, cough, and hoarseness, showed advantages in the LMA group, which were reflected in the lower incidence and severity. The perioperative pain in the patients was also effectively managed, as the patients had better subjective feelings, and the satisfaction degree with LMA was also higher. Furthermore, as the LMA was not implanted in the airway, the injuries on pharyngeal and tracheal mucosa were reduced, and the incidence of postoperative complications such as pharyngalgia, edema, and hoarseness was lower.^17^, ^18^ Especially in young patients, the postoperative symptoms were less severe, thus having a lower influence on the socialization after discharge and helping the patients to rid of the “patients” identity as early as possible and return to their preoperative state.

Regarding the effectiveness endpoints, the findings of this study showed that the satisfaction degree with the anesthesia and surgical fields did not significantly differ between the LMA and ETT groups. After the LMA was completed, the thoracic cavity was accessed, an open pneumothorax was induced, and the lung tissue collapsed, which provided a relatively adequate operation field for thoracoscopic procedures.^12^ The key step was to reduce the breathing movement, thus increasing the field for the thoracoscopic surgery, which could be achieved by temporarily stopping the ventilation or adding the right dose of opioids to reduce the respiration rate at the key step of surgery.^12^ Of course, these procedures required close cooperation between anesthetists and operators. Previous studies^9^, ^18^ reported that the operation time of thoracoscopic surgeries under LMA was increased to a certain extent, which could be associated with the requirement for cooperation between anesthetists and operators to adjust the respiratory parameters, as well as the techniques and skillfulness of operators. Our results showed that when the anesthetists cooperated well with operators, the operation time did not significantly differ between the LMA and ETT groups. From the aspect of safety endpoints, the findings of this study showed that intraoperative blood loss and lowest SPO_2_ were not significantly different between the two groups. The highest end‐tidal partial pressure of CO_2_ was higher in the LMA group than in the ETT group in this study. LMA could increase the respiratory dead space of patients and consequently lead to the accumulation of CO_2_ in the body; however, it has been well acknowledged that permissive hypercapnia can prevent lung injuries induced by high tidal volume and over ventilation.^19^ During surgical procedures, the end‐tidal carbon dioxide partial pressure can be continuously monitored, after which intermittent auxiliary manual low tidal volume ventilation can be performed to promote the discharge of excess CO_2_. Blood gas parameters were re‐examined 1 h after the operation and were not significantly different between the two groups, indicating that the ventilation functions of patients could rapidly recover. Our findings also showed that the anesthesia recovery time and postoperative hospital stay were substantially better in the LMA group than in the ETT group. As bronchial intubation substantially stimulates the cardiovascular system, relative deep anesthesia and muscle relaxation are generally required.^20^ The doses of anesthetic agents in the LMA group were substantially lower than in the ETT group, and the patients recovered from anesthesia faster, which allowed them to restore their respiratory function and perform out‐of‐bed activities rapidly. Consequently, this also facilitated intestinal tract movement, promoted recovery of the digestive system, reduced postoperative gastrointestinal discomfort, and shortened the postoperative hospital stay.^9^ Therefore, LMA is in agreement with the advancement of the ERAS concept. However, previous studies^9^, ^17^, ^20^ have also reported that LMA also involves the risks of reflux, aspiration, and air leakage and is incapable of isolating the lungs. Therefore, these indications must be carefully evaluated, and conversion to endotracheal intubation should be immediately performed if necessary.

The sample size of this study was relatively small, and double‐blind could not be applied due to the clinical diagnosis and treatment. More high quality, multicenter, randomized controlled trials with large sample sizes are needed to verify the findings of this study.

In conclusion, LMA is an effective and safe anesthesia method that can be applied in thoracoscopic surgeries. It promotes postoperative recovery, improves postoperative symptoms, and shortens the hospital stay. Therefore, LMA is an important component of ERAS strategy in thoracic surgery departments, which may have more substantial advantages in relatively simple surgeries, such as palmar hyperhidrosis, NUSS surgery, and wedge pneumonectomy.

## CONFLICT OF INTEREST

The authors declare that they have no conflict of interest.
